# *o*-carborane encapsulated into polymeric micelles for proton boron capture therapy

**DOI:** 10.1186/s12645-026-00400-2

**Published:** 2026-06-28

**Authors:** Guodong Zhang, Qiu-Xu Teng, Célia Fernandes, Haiyan Chen, Filipa Mendes, Narayan Sahoo, Ping Pan, Lurdes Gano, António Paulo, Chun Li

**Affiliations:** 1 Departments of Cancer Systems Imaging - Unit 1907, The University of Texas MD Anderson Cancer Center, 1881 East Road, Houston, TX 77054, USA; 2 Department of Nuclear Sciences and Engineering, Center of Nuclear Sciences and Technologies, Instituto Superior Técnico, University of Lisboa, Estrada Nacional 10, Km 139.7, Bobadela LRS 2695-066, Portugal; 3 Radiation Physics, The University of Texas MD Anderson Cancer Center, Houston, TX 77054, USA

**Keywords:** Proton therapy, Proton boron capture therapy, o-Carborane, Polymeric micelles, Pancreatic ductal adenocarcinoma

## Abstract

Proton boron capture therapy (PBCT), based on proton-boron fusion reactions (^11^B + p →3α + 8.7 MeV), has the potential to enhance the biological effectiveness of proton therapy. To achieve this potential, the efficient delivery of enough ^11^B to cancer cells will be paramount. This study demonstrates a newly developed method of poor solvent–mediated spontaneous assembly of polymeric micelles with entrapped *o*-carborane, in which ethanol is a good solvent for the payload *o*-carborane but a poor solvent for the hydrophobic segment poly(ε-caprolactone) in this amphiphilic block copolymer. This method provided smaller polymeric micelles compared to use of tetrahydrofuran, a good solvent for the amphiphilic block copolymer, as the sole solvent for both payload and copolymer. The prepared nanoscale formulation enabled the boron-rich cage compound *o*-carborane to be effectively delivered to tumor cells, and its enhancement of the biological effectiveness of proton therapy was preliminarily validated in a pancreatic ductal adenocarcinoma cell line, MiaPaCa-2, by survival assays and DNA damage assessment. The ^111^In-labeled congener was also successfully prepared and used in biodistribution assays in normal mice, opening the way to further studies on image-guided PBCT in preclinical animal models.

## Introduction

Proton therapy is one of the most advanced and precise modalities of radiotherapy for cancer treatment due to the unique depth-dose characteristics of proton beams. The dose deposition of proton beams in tissue is well defined. The absorbed dose sharply increases at a specific depth that is dependent on the initial beam energy, called the Bragg peak, and then drops sharply within a limited distance beyond the Bragg peak region. These characteristics of proton beams lead not only to high dose deposition in tumor tissue but also to a 50% to 70% lower dose to healthy tissue around the tumor (Yan et al. 2023). Proton therapy has been applied to the treatment of pediatric neoplasms, head and neck tumors, hepatocellular carcinoma, and gastrointestinal malignancies. Despite its advantages, proton therapy has a moderate relative biological effectiveness (RBE) value of ~ 1.1, compared to α particle and carbon ion beams, which have RBE values as high as 2.0 and 3.0, respectively (Luhr et al. 2018). Thus, increasing the RBE of proton therapy is of great interest, with the goal to extend the benefits of proton therapy to more cancer patients.

Proton boron capture therapy (PBCT) has exhibited the potential to increase proton therapy’s RBE. PBCT is based on the nuclear fusion reaction of protons with boron-11 (^11^B), which makes up 80% of natural boron, to yield α particles. Using Monte Carlo simulations, Yoon et al. (Yoon et al. 2014) first proposed the idea of using the proton-boron fusion reaction, ^11^B + p →3α (total 8.68 MeV), to generate α particles to enhance cell killing. In 2018, Cirrone et al. (Cirrone et al. 2018) reported the first experimental proof of PBCT by using sodium borocaptate (BSH) to significantly enhance proton therapy’s cancer cell killing ability in a human DU145 prostate cancer cell line. The cells were irradiated with a 62 MeV proton beam after treatment with BSH. Recently, the concept of PBCT was extended to several other tumor cell lines including U-87 MG glioblastoma cells. Cells were irradiated with 190 MeV proton beams in Bragg peak regions. The authors reported a 2- to 3-fold reduction in cell survival in the presence of 40 ppm natural boron in culture media in the form of BSH (32 ppm ^11^B), validating the effectiveness of PBCT (Jelínek Michaelidesová et al. 2024). A first preclinical evaluation of PBCT in a subcutaneous glioblastoma model showed induction of significantly reduced glycolysis and increased apoptosis by PBCT compared to proton therapy alone (Cammarata et al. 2023). In that study, boron was delivered by a single intraperitoneal injection of 500 mg/kg of boronophenylalanine (BPA) in water. These in vitro and in vivo data suggest that boron-containing compounds could increase RBE of proton beams.

However, the effectiveness of PBCT and its physical and biological effects remain controversial (Hosobuchi et al. 2023, Manandhar et al. 2022, Tran et al. 2023, Shtam et al. 2023). For example, Manandhar et al. (Manandhar et al. 2022) found that ^11^B at concentrations of 80 ppm from BSH or 87 ppm from BPA had no biological effect in DU145 prostate cancer cells after irradiation with proton beams. Shtam et al. (Shtam et al. 2023) observed no significant enhancement in cell killing when several tumor cell lines, including the previously studied DU145, were incubated with BSH prior to proton beam irradiation. Of relevance to the evaluation of effectiveness of PBCT, in all these studies, the cellular concentration and tumor uptake of boron are not known. This gap underlines the need to better investigate the effects of the cellular uptake of the boron-containing compounds and of treatment conditions on the enhancement effect of PBCT for proton therapy.

To properly evaluate PBCT, effectively delivering sufficient boron compounds to tumor cells before proton irradiation is needed. Targeted delivery of boron-containing drugs to cancer cells, both in vitro and in vivo, has been studied with boron neutron capture therapy (BNCT), in which ^10^B-containing compounds are selectively delivered to tumor cells. When irradiated with neutrons, such compounds can yield highly energetic α particles to destroy tumor cells. BNCT with ^10^B-BPA has been approved for treatment of recurrent or locally advanced head and neck cancer in Japan since 2021 (Sato et al. 2024). In BNCT, effective boron concentrations of 10 ~ 30 μg per gram of tumor tissue were achieved with BPA through the amino acid transporter LAT1, which is highly expressed in head and neck tumor cells. Yet, for tumors that express low levels of LAT1, such as pancreatic tumors, alternative efficient delivery methods are needed. Several groups have reported targeted delivery of boron-containing compounds, namely glucose-conjugated BSH (Fujimoto et al. 2024), transactivator of transcription (TAT) peptide (GRKKRRQRRRPQ)-conjugated carborane decorated with hyaluronic acid (Quan et al. 2021), 1-to-3 arginine peptide-conjugated BSH (Iguchi et al. 2015), polymer-conjugated BPA (Nomoto et al. 2021, Nomoto et al. 2020), liposomes, and polymeric micelles (Li et al. 2022).

Polymeric micelles have shown great potential in the delivery of hydrophobic drugs for cancer treatment. Compared to liposomes, polymeric micelles offer advantages such as small size (20–100 nm in diameter); relatively high drug loading (up to 50% by weight) (Cabral et al. 2018, He et al. 2016, Serras et al. 2024); codelivery of hydrophobic chemotherapeutic drugs, even three-in-one combination therapies (Shin et al. 2012, Zhao et al. 2018); multiple choices of preparation methods for different drug/polymer systems, including film-rehydration and microfluidic nanoprecipitation (Serras et al. 2024), and proven scaling-up technologies for translational development and production (Ghezzi et al. 2021). Moreover, an array of methods—such as atom-transfer radical polymerization (ATRP), reversible addition–fragmentation chain transfer (RAFT) polymerization, ring-opening metathesis polymerization (ROMP), and controlled ring-opening polymerization of cyclic esters and *N*-carboxyanhydrides—make a variety of amphiphilic copolymers available for building polymeric micelles (Cabral et al. 2018). To date, more than 20 polymeric micellar formulations of chemotherapeutic drugs have entered clinical trials, and at least 4 polymeric micelles are marketed in the oncology space (Serras et al. 2024). Marketed products include Genexol PM (2007, active drug paclitaxel, marketed in Korea), Nanoxel (2007, active drug paclitaxel, marketed in India), Nanoxel-PM (2012, active drug paclitaxel, marketed in Korea), and PM-PTX (2021, active drug paclitaxel, marketed in China). Brush-like copolymers tend to show lower critical micelle concentrations (CMCs) than congener linear block copolymers, facilitating micelles formation and more efficient drug encapsulation. Using a newly synthesized brush-like amphiphilic copolymer, Zhao et al. (Zhao et al. 2018, Zhao et al. 2018) developed a polymeric micellar system for co-delivery of 2 drugs for the treatment of pancreatic ductal adenocarcinoma (PDAC).

In this report, we utilized this brush-like amphiphilic block copolymer to entrap a boron-rich cage compound, ortho-carborane (*o*-carborane), to prepare a new polymeric micelle, termed M-Carb, for evaluating PBCT. Our objective was to achieve an efficient micellar encapsulation of the boronated compound for its effective delivery to PDAC cancer cells aiming at their radiosensitization within PBCT therapeutic approaches. In contrast to conventional micelle-formation methods in which a good polymer solvent is used for dissolution of both the amphiphilic block copolymer and the drug payload, we found that the use of a poor polymer solvent for drug dissolution could markedly impact the size of the resulting drug-loaded micelles. Moreover, we showed that micelles loaded with *o*-carborane (i.e., M-Carb) could sensitize PDAC cells to proton therapy. In addition, the labeling of M-Carb with ^111^In allowed dual biodistribution studies in normal mice, based on gamma counting measurements and analytical determination of boron concentrations in selected organs and tissues. Our results support the use of boron compounds for PBCT and open the way to image-guided PBCT approaches with the new boron-containing polymeric micelle.

## Results and discussion

### Polymeric micelles encapsulating o-carborane M-Carb displayed favorable physicochemical properties

Poly[oligo(ethylene glycol) monomethyl ether methacrylate]_21_-*b*-poly[2-hydroxyethyl methacrylate-*g*-(ε-caprolactone)_9_-mono-succinate ester)]_22_ (**P4**) was used to generate polymeric micelles entrapped with *o*-carborane as the ^11^B source for the proton-boron fusion reaction. *o*-Carborane (C_2_H_12_B_10_) is soluble in organic solvents such as tetrahydrofuran (THF) and ethyl alcohol, making it a suitable drug for micelle encapsulation with **P4**. We have anticipated that the presence of the terminal carboxylic group in **P4** could promote favorable intermolecular interactions with o-carborane, stabilizing the micellar core and favoring its entrapment, namely through the formation of hydrogen bonds involving the weakly acidic CH protons in the carborane cage.

The block copolymer used in the M-Carb micelle formulation was synthesized by ATRP. Subsequent ring-opening polymerization of ε-caprolactone and end capping of the hydroxyl groups by succinates in the hydrophobic segment yielded **P4** (Fig. 1). The degrees of polymerization of the hydrophilic segment and the hydrophobic segment were 21 and 22, respectively. The number of repeating units of the grafted oligo(ethylene glycol) in the hydrophilic segment was 9, and the number of repeating units of the grafted oligo(caprolactone) in the hydrophobic segment was 9.

To prepare the *o*-carborane-entrapped polymeric micelle, water was slowly added to the polymer/drug solution in water-miscible organic solvents. Here, THF was used in the preparation of **P4** polymer solution (100 mg/mL), and ethanol or THF was used for the preparation of the *o*-carborane solution. Conventionally, in the nanoprecipitation method, an organic phase containing polymer and drug payload is added to an aqueous phase, leading to rapid phase separation and formation of nanoparticles. We found that reversing the order of mixing the organic and water phases yielded micelles with optimal physicochemical characteristics. Thus, water (2-fold the volume of THF/polymer solution) was slowly added to the polymer/payload solution at the rate of 0.5 mL/min under vigorous magnetic stirring. After removal of the organic solvent by rotary evaporator, polymeric micelles in water were purified by centrifugation to remove solid impurities including unentrapped drugs, followed by tangential flowing filtration to completely remove residual organic solvent.

The effects of the organic solvents used for dissolving polymer and *o*-carborane, polymer-to-carborane weight ratio, and polymer concentration were investigated, and the data are summarized in Table 1. For this study, we have considered “good polymer solvents” (i.e., favoring solvent-polymer over polymer-polymer interactions with expansion of polymer chains and enabling dissolution) and “poor polymer solvents” (i.e., favoring polymer-polymer over solvent-polymer interactions with polymer chains contraction and precipitation). THF is a good solvent for both polymer and *o*-carborane. When THF was used to dissolve both **P4** and *o*-carborane, the resulting *o*-carborane-loaded polymeric micelles (M-Carb-6a) had an average diameter of 132 nm. When *o*-carborane was dissolved in ethanol, which is a poor solvent for poly(ε-caprolactone) (Lim et al. 2008), mixing *o*-carborane in ethanol with **P4** in THF at a THF-to-ethanol volume ratio 1:0.24 to 1:0.48 yielded micelles with a smaller diameter of 72 to 76 nm (M-Carb-6, M-Carb-12, M-Carb-6b, and M-Carb-12a) (Table 1; Fig. 2A). This result suggests that the oligo(ε-caprolactone) chains grafted to the hydrophobic block of the copolymer collapsed together with *o*-carborane in the presence of ethanol to form a tight micellar core when water was added to the organic solution. Thus, the organic solvents used to dissolve polymer and drugs play a critical role in the formation of polymeric micelles. In addition to the smaller nanoparticle size, the entrapment efficiency was higher with the THF/ethanol solvent system than with THF alone for the polymer/drug solutions (65.3% for M-Carb-6 vs. 58.0% for M-Carb-6a) (Table 1). Decreasing the polymer concentration from 100 mg/mL to 50 mg/mL resulted in decreased entrapment efficiency (65.3% for M-Carb-6 vs. 56.3% for M-Carb-6b, and 57.5% for M-Carb-12 vs. 52.8% for M-Carb-12a). All M-Carb preparations displayed negative charge, with zeta potentials ranging from about − 8.0 mV to − 9.1 mV (Table 1). The highest *o*-carborane payload in M-Carb-12 achieved was 6.45% (w/w), which is significantly higher than the reported value of < 2.0% in other micellar systems (Meher et al. 2021).

Critical micelle concentration was determined by directly measuring the dynamic light scattering intensity of M-Carb after serial dilution in phosphate-buffered saline (PBS) (Fig. 2B). The critical micelle concentration of M-Carb-12 was estimated to be ~ 10 μg P4/mL. Figure 2C shows the drug release profile of M-Carb-12 at 37 °C in PBS containing 10% fetal bovine serum (FBS). About 25% of Carb was released in the first 4 h, and 80% was released over a 24 h period. At this point, we should clarify that for PBCT the release of the boronated compound from the carrier micelles is not necessary, as the proton-boron fusion reaction also occurs inside the micelles and the generated α particles can escape the micelles to hit the DNA of the tumor cells. In all of the subsequent studies, M-Carb-12 was used, and it is referred to as M-Carb hereafter.

### M-Carb displayed cytotoxicity similar to BPA and was taken up by MiaPaCa-2 PDAC cells

As an initial biological characterization step, the cytotoxicity of M-Carb and, for comparative purposes, of BPA was assessed using the CCK-8 assay in 3 cell lines: the human MiaPaCa-2 PDAC cells, murine Kras* cells with an inducible Kras^G12D^ mutation, and human pancreatic stellae cells (HPSCs) representing normal cells. Treatment with either compound led to a dose-dependent reduction in cell viability in the 3 cell lines. For M-Carb at the highest concentration (1 mM) tested, 68.9 ± 5.0% MiaPaCa-2 cells remained viable, 58.8 ± 1.4% Kras* cells remained viable, and 73.3 ± 3.2% HPSCs remained viable. A similar trend was observed with BPA (Fig. 3).

Next, the cellular uptake of M-Carb was studied in human MiaPaCa-2 PDAC cells. Cells were incubated for 6 h with micelles with 0.2 mM and 1.0 mM concentrations of *o*-carborane, and for comparative purposes with BPA at the same molecular concentration. After incubation and washing, the boron content in cell pellets was quantified by inductively coupled plasma mass spectrometry (ICP-MS). For M-Carb at 0.2 mM and 1.0 mM of *o*-carborane, the cellular boron uptake in MiaPaca-2 cells was 41.84 ± 11.10 and 53.18 ± 11.31 ng/million cells, respectively. In comparison, the cellular boron concentrations in cells incubated with BPA were 1.60 ± 0.11 and 18.85 ± 1.22 ng/million cells, respectively, being significantly lower than those achieved with M-Carb (Fig. 3B).

### M-Carb reduced cell survival in a radiation dose-dependent manner

After confirming that M-Carb was able to deliver boronated compounds to MiaPaCa-2 cells, we investigated the radiosensitizing effects of M-Carb in comparison with BPA using the colony formation assay. Cells were incubated with M-Carb or BPA for 6 h and then irradiated with a 100 MeV clinical proton beam. The irradiation studies were performed at a 5 Gy dose with protons from the entrance (1.22 cm, low linear energy transfer [LET]) and distal sides (4.4 cm, high LET) of the Bragg peak. Results showed that the presence of boronated compounds enhanced cell killing after irradiation with a 5 Gy dose, but only with the high-LET proton beam, and that M-Carb but not BPA significantly reduced the fraction of surviving cells when compared to radiation alone (Fig. 4A).

Next, we investigated the response of MiaPaCa-2 cells to proton irradiation in the presence and absence of either M-Carb or BPA with a high-LET proton beam at doses ranging from 1 Gy to 8 Gy. A clonogenic assay revealed that both BPA and M-Carb reduced cell survival in a radiation dose-dependent manner (Fig. 4B). Notably, cells pre-treated with M-Carb exhibited significantly fewer colonies than did those treated with radiation alone (control) at 1 Gy, 2 Gy, and 8 Gy. BPA pretreatment also led to fewer colonies formed compared to radiation alone (Fig. 4C). However, the reduction in survival fractions was statistically significant only with M-Carb (*p* < 0.05) (Fig. 4D). The survival results were fitted into the linear quadratic regression model to estimate α and β parameters (Table S1). The α parameter indicates the probability of a single ionizing event resulting in a DNA double-strand break (DSB). The α parameter of cells incubated with the boronated compounds were higher than those of the controls (control, 0.109 ± 0.103; BPA 0.251 ± 0.113; M-Carb, 0.302 ± 0.122), confirming a radiosensitizing effect. These experimental data confirmed the effectiveness of PBCT provided that a sufficient quantity of boron-containing compounds is taken up by tumor cells.

### BPA or M-Carb potentiated DNA damage by the proton beam

γ-H2AX and 53BP-1 are markers for DNA DSBs. γ-H2AX is a phosphorylated form of the histone protein H2AX. It forms foci at the sites of DSBs within the cell nucleus. The damage sensor p53-binding protein 1 (53BP1) also accumulates at DNA damage sites associated with the chromatin surrounding DSBs (Shibata and Jeggo 2020, Scully and Xie 2013). We used both biomarkers to assess DNA damage in MiaPaCa-2 cells following treatment with BPA or M-Carb and proton beam irradiation (Fig. 5). Analysis of the number of γ-H2AX foci showed that (1) γ-H2AX-positive foci increased significantly with increasing radiation dose; (2) both BPA and M-Carb significantly increased the number of γ-H2AX-positive foci compared to the corresponding controls without pretreatment (i.e., 0 Gy, 2 Gy, and 5 Gy distal-end proton irradiation); (3) cells treated with BPA or M-Carb alone had significantly more γ-H2AX-positive foci compared to the no-treatment and no-radiation controls; and (4) M-Carb induced significantly more DNA DSBs than BPA when combined with 2–5 Gy proton beam irradiation (Fig. 5B).

Similarly, 53BP1-stained MiaPaCa-2 cells treated with M-Carb and proton irradiation exhibited significantly greater immunofluorescence signal intensity than did cells treated with radiation alone. Moreover, M-Carb in combination with 2–5 Gy proton beam irradiation induced significantly higher fluorescence signal intensity of 53BP1-stained cells than did the combination of BPA with the same doses of proton radiation (Fig. 5C). These data indicate that both BPA and M-Carb sensitized PDAC cells to proton beam radiation, and that M-Carb was more potent than BPA in its radiosensitizing effect. Giving our finding that M-Carb mediated significantly higher cellular uptake of boron, the observed difference in potency could be explained by higher cellular boron concentration.

### ^111^In-labeled M-Carb demonstrated prolonged blood radioactivity

The M-Carb micelles were labeled with ^111^In (T_1/2_ =2.83 d), a commercially available gamma-emitting radionuclide widely used to assess micelle biodistribution in either ex vivo or imaging studies due to its favorable half-life and radiolabeling chemistry (Laan et al. 2016). The labeling strategy involved the incubation of preformed M-Carb micelles with ^111^In-oxine, a small and lipophilic In(III) complex able to diffuse into the micellar inner core (Fig. 6A) (Fuente et al. 2019). As detailed in the experimental section, the radiolabeled micelles (^111^In-M-Carb) were recovered by ultrafiltration in high radiolabeling yield (ca. 90%), as verified by instant thin-layer-silica gel radiochromatography. ^111^In-M-Carb presented high in vitro stability, as no radioactivity was released from the micelles after 24 h in solution at 37 °C (Fig. S1), showing that stable radiolabeling was achieved.

The biodistribution of ^111^In-M-Carb was assessed in a CD1 mouse model. ^111^In-M-Carb was injected into the tail vein. At 1–24 h post-injection, a group of animals were euthanized and dissected. Major organs and samples of tissues were collected and rinsed, and the radioactivity of ^111^In was measured with a gamma counter to calculate the percentage of radioactivity taken up by different organs. To assess the level of boron retained in the main organs, the concentration of boron in tissues was determined by ICP-MS analysis after ^111^In activity was completely decayed.

Data were expressed as a percentage of injected activity and are presented in Fig. 6B. The results indicated that approximately 15% of the injected activity remained in the bloodstream for up to 24 h after administration and that radiolabeled M-Carb was mainly taken up by the excretory organs, i.e., the liver and kidney, and to a lesser extent the intestine. The level of total radioactivity excreted from the animal body was lower than 20%. ICP-MS analysis of boron content in the same organs confirmed the presence of a measurable amount of boron in the blood at 1 h after injection; the highest boron accumulation was found in the liver and the kidney, consistent with the radioactivity counting data (Table S2). However, we should mention that the boron atoms detected in the tissues and organs can arise from free or encapsulated o-carborane, with the former expected to have a faster rate of excretion with possible reduction of tumor accumulation. Due to the lower sensitivity of ICP-MS analysis compared to gamma counting measurements, the boron content of other organs was below the threshold detection limit of ICP-MS and could not be ascertained.

## Conclusions

We prepared M-Carb by a newly developed polymeric micelle formation method that used a poor solvent for polymer to dissolve *o*-carborane, resulting in smaller nanoparticles than those produced by a method using a good polymer solvent to dissolve *o*-carborane. M-Carb was efficiently taken up by MiaPaCa-2 cells and achieved significantly higher intracellular boron accumulation than BPA did. PBCT mediated by M-Carb and BPA enhanced the ability of proton therapy to kill PDAC cells under high-LET conditions, probably because the α particles emitted caused more irreparable DNA damage in cancer cells. Boron-rich molecular cage compounds such as carboranes demonstrated the advantage of enhanced cellular delivery with polymeric micellar delivery system. M-Carb with [^10^B]*o*-carborane may also be suitable for BNCT.

M-Carb was successfully labeled with ^111^In to produce ^111^In-M-Carb, which exhibited high in vitro radiochemical stability. Biodistribution studies of ^111^In-M-Carb in normal mice showed that the radiolabeled micelles have slow blood clearance and are mainly taken up by the excretory organs. Further studies to demonstrate the potentiation of antitumor effects of PBCT with M-Carb in preclinical animal models of PDAC are warranted.

## Experimental section

### Materials

4-Boron-L-phenylalanine (BPA) and anhydrous tetrahydrofuran (THF) were obtained from Thermo Fisher Scientific (Asheville, NC); *o*-carborane (C_2_B_10_H_12_) was purchased from Synthonix (Wake Forest, NC). D-fructose was obtained from TCI America (Portland, OR). All other solvents and chemicals were purchased from Sigma-Aldrich (St. Louis, MO).

Dulbecco’s modified Eagle’s medium (DMEM), DMEM/F12 (a 50:50 mix of DMEM and Ham’s F-12), FBS, penicillin/streptomycin, 0.25% trypsin, and 1X PBS were obtained from the Media Facility at The University of Texas MD Anderson Cancer Center. The anti-phospho-histone H2AX (Ser139,20E3) antibody (γH2AX staining kit) was purchased from Abcam (Waltham, MA). A 53BP1 (clone E7N5D) rabbit mAb was sourced from Cell Signaling Technology (Danvers, MA). DAPI solution, mounting medium, and Alexa Fluor 488- or 594-conjugated goat anti-rabbit secondary antibodies were acquired from Invitrogen (Waltham, MA).

Block copolymer poly[oligo(ethylene glycol) monomethyl ether methacrylate)_21-_*b*-poly[2-hydroxyethyl methacrylate-*g*-(ε-caprolactone)_9_-mono-succinate ester)]_22_ (**P4**) was prepared according to a previously reported method (Zhao et al. 2018). Briefly, block **P4** was synthesized via sequential atom transfer radical polymerization of oligo(ethylene glycol) monomethyl ether methacrylate (PEGMA) and 2-hydroxyethyl methacrylate (HEMA), which was followed by ring-opening polymerization of ε-caprolactone to introduce oligo(ε-caprolactone) at the hydroxy groups of the HEMA block of the copolymer. The terminal hydroxyl groups of oligo(ε-caprolactone) were converted to carboxylate by reacting with succinic anhydride in the presence of dimethyl aminopyridine catalyst to yield **P4** (Fig. 1A).

### Preparation of BPA-fructose complex solution

BPA was mixed with fructose solution in double-distilled water at a molar ratio of 1:1.5, and pH was adjusted to 10.5 by adding 1.0 M NaOH under stirring during BPA solubilization. Thirty minutes after BPA was completely dissolved, pH was adjusted to 7.6 with 1.0 M HCl. The solution was sterile filtered using a 0.22 μm syringe filter.

### Micelle preparation and characterization

*o*-Carborane solution in THF or ethanol (conc. 25 mg/mL) was added to a solution of **P4** in THF (100 mg/mL). The mixture was stirred for 10 min to achieve complete dispersal. Then, 2X volume of deionized water was added dropwise to the organic solution under vigorous magnetic stirring for 2 min. The organic solvent was removed by rotary evaporator, and the micelle dispersion in water was subjected to centrifugation (10 K RPM) for 10 min to remove unencapsulated drug. The supernatant was collected and filtered through a 0.22 μm syringe filter. The resulting micelles were stored at 4 °C for further use.

For characterization of the drug-loaded polymeric micelles (M-Carb), the boron payload in the nanoparticles was quantified using inductively coupled plasma optical emission spectrometry (ICP-OES (Perkin Elmer Avio 550) after dilution with ICP matrix. Samples for ICP-OES analysis were prepared by dissolving M-Carb-treated cell pellets in concentrated H_2_SO_4_ and HNO_3_ (1:1, v/v), diluted in deionized water containing 0.3% Triton X-100. Micelle size was measured by Zetaplus (Brookhaven) in PBS solution (1.0 mg/mL). Surface charge was measured by a zeta sizer on Litesizer 500 (Anton Paar), 1.0 mg/mL in 10 mM PBS. Critical micelle concentration was determined by the dynamic light scattering method, which measures the change in intensity of scattered light of different concentrations of micellar dispersion. *o*-Carborane released from M-Carb was analyzed by a dialysis membrane method. In brief, M-Carb was diluted to 0.2 mg of *o*-carborane/mL PBS, and 100 μL of diluted M-Carb was added to a Slide-A-Lyzer mini dialysis unit (10 K MWCO, PIERCE). The dialysis disks were left in a 50-mL beaker containing 45 mL of releasing medium (10% FBS in pH 7.4 PBS) with gentle shaking at 37 °C. At 0.5, 1.5, 3, 6, 12 and 24 h, 10-μL aliquots were collected from the dialysis disks and diluted to 3 mL with the ICP matrix. Boron concentrations remaining in the dialysis disks were quantified using ICP-OES.

### Micelle radiolabeling

^111^In-oxine was prepared by mixing ^111^InCl_3_ in acetate buffer, pH 5.0, with 8-hydroxyquinoline (oxine) in ethanol. After 5 min at room temperature the ^111^In-oxine complex was extracted into methylene chloride, which was completely evaporated to dryness. The o-carborane loaded polymeric micelles (M-Carb) were added to the dry residue of ^111^In-oxine and the solution was vortexed and incubated at 37 °C for 20 min (Fig. 6A). Then, radiolabeled micelles ^111^In-M-Carb were purified using 10 kDa Amicon centrifugal filters to remove the non-encapsulated ^111^In-oxine. The purified radiolabeled micelles ^111^In-M-Carb were obtained with high radiolabeling yield (ca. 90%). The in vitro stability of ^111^In-M-Carb was tested for up to 24 h in PBS solution pH 7.4 at 37 °C, with instant thin layer chromatography analysis using sodium acetate buffer pH 5 as eluent (Fig. S1).

### Cell lines and cell culture

The human pancreatic carcinoma cell line MiaPaCa-2 (Catalog CRM-CRL-1420, RRID: CVCL_0428) was obtained from ATCC and cultured in DMEM supplemented with 10% FBS and 1% penicillin-streptomycin. Murine pancreatic cancer Kras* cells with a doxycycline-inducible KRAS^G12D^ (RRID not applicable) mutation were kindly provided by Dr. Ronald A. DePinho at The University of Texas MD Anderson Cancer Center. The cells were cultured in DMEM/F12 medium supplemented with 1 μg/mL doxycycline. Kras* cells were used in this study only for cytotoxicity assessment, but were not used in the radiosensitization study with proton beam irradiation, and the use of the cell line does not have an effect on the conclusions. Human pancreatic stellate cells (HPSCs) (RRID: CVCL_SA58) were kindly provided by Dr. Rosa F. Hwang (MD Anderson Cancer Center) and were maintained in DMEM/F12 medium (Hwang et al. 2008). All cell lines were incubated at 37 °C in a humidified atmosphere containing 5% CO_2_.

### Cytotoxicity assay

MiaPaCa-2, Kras*, and HPSC cells were seeded at a density of 1000 cells per well in 96-well plates and allowed to attach for 24 h. The cells were then treated with BPA or M-Carb at varying concentrations and incubated for an additional 72 h at 37 °C. Cell viability was evaluated using the Cell Counting Kit-8 (CCK-8) from GLPBIO (Montclair, CA) following the manufacturer’s instructions.

### Cellular boron uptake and intracellular boron retention

To evaluate cellular uptake of boron, MiaPaCa-2 were seeded on a dish (5 × 10^6^ cells per dish) and incubated for 24 h. Cells were incubated with M-Carb or BPA for 6 h in fresh culture medium at equivalent drug concentrations of 0.2 mM or 1.0 mM. The cells were collected, washed, and counted with a TC20 automated cell counter (Bio-Rad). After digestion in 400 μL of 1:1 (v/v) mixture of concentrated H_2_SO_4_/HNO_3_ for 24 h, samples were diluted to 10 mL using ICP matrix and filtered using a 0.22 μm syringe filter. The amount of boron in samples was quantified using ICP-MS (Agilent 7900 ICP-MS, Agilent Technologies Inc., Santa Clara, CA). The intracellular boron concentration (^11^B ng/10^6^ cells) was calculated from the amount of boron measured and the cell number.

### Proton beam irradiation

Cells were irradiated in a passive scattering proton beam of a Hitachi ProBeat machine (Hitachi, Ltd., Tokyo, Japan) at the MD Anderson Cancer Center Proton Therapy Center. An 18 cm × 18 cm unmodulated 100-MeV proton beam with a practical range (the depth of the 10% of Bragg peak dose) of 4.5 cm (74 MeV proton beam at the entrance based on the continuous slowing down approximation (CSDA) range table for liquid water from Proton STopping power and Range Tables (PSTAR) of the US National Institute of Standards and Technology (Berger et al. 2005). The cells were placed at water-equivalent depths of 1.2 cm at the proximal side and 4.4 cm at the distal side of the Bragg peak. At 1.2 and 4.4 cm, the Fluence-weighted average LET values in water were approximately 1.0 and 7.5 keV/μm, respectively (McFadden et al. 2016). The energies of the proton beam at these depths are estimated to be 62 and 9 MeV based on their CSDA range at these depths, respectively. Cells were irradiated in 6-well plates or 8-well chamber slides. The plates or slides were irradiated with the gantry at 180°, so that proton beam passed though the treatment couch, water-equivalent plastic blocks, and the bottom of the plates or slides to a combined water-equivalent depth of 1.2–4.4 cm. The difference in the thickness of the 6-well plates and 8-well chamber slides was considered in choosing the thickness of the plastic water placed under them so that the cells would be irradiated at the same depths for both containers. Cells placed at the 1.2 cm depth were exposed to a single 5 Gy dose and those at 4.4 cm were exposed to doses ranging from 1 to 8 Gy. A fixed source-to-surface distance of 270 cm was used in the irradiation. The dose per monitor unit (MU) was measured at different depths using a calibrated parallel plate ion chamber following the International Atomic Energy Agency (IAEA) Technical Report Series 398 (TRS-398) protocol (Andreo et al. 2006), and the MUs were calculated using the measured dose/MU values. The dose uncertainties arising from cell positioning or beam fluctuation were estimated as being within ± 2%.

### Colony formation assay

The clonogenic assay assessed the effects of BPA and M-Carb under different radiation conditions. In the first experimental set, MiaPaCa-2 cells were seeded at densities of 100 or 300 cells per well in 6-well plates and allowed to adhere overnight. Cells were then incubated with 40 ppm of ^11^B in BPA or M-Carb for 6 h, followed by exposure to either 5 Gy entrance or 5 Gy distal-end proton beam irradiation. In the second set, cells were seeded at densities of 100, 300, or 500 cells per well, treated with 40 ppm of ^11^B in BPA or M-Carb for 2 h, and subjected to 1 Gy, 2 Gy, 5 Gy, or 8 Gy distal-end proton irradiation. Control cells were incubated only with culture medium and irradiated (irradiation control) or with compounds and not exposed to radiation (non-irradiated control).

After irradiation, cells were incubated in fresh medium for 14 days to allow colonies to form. Colonies were fixed with methanol: crystal violet (3:1), photographed, and the number of colonies with sizes > 50 μm were manually counted. Survival fraction (SF) was calculated as: SF = plating efficiency of cells treated with drugs with or without radiation / plating efficiency of cells treated with PBS only (no drug and no radiation). Plating efficiency (PE) was calculated as PE = number of colonies counted / number of cells plated.

For the survival curves, a fitting of the linear quadratic regression model was performed with GraphPad Prism software: SF = e^−αD–βD2^. in which D corresponds to the dose, α corresponds to the probability of a single ionizing event resulting in a DNA DSB, which was considered lethal damage, and β corresponds to the probability of multiple ionizing events to cause a set of DNA single-strand breaks that, combined, result in DSBs, which was considered sublethal damage (McMahon 2018).

### Immunofluorescence assay

MiaPaCa-2 cells were seeded at a density of 500 cells per chamber in 8-well chamber slides and incubated overnight. After incubation with 40 ppm ^11^B of BPA or M-Carb for 6 h, cells were exposed to 0 Gy (non-irradiated control), 2 Gy, or 5 Gy distal-end proton irradiation. Control cells were incubated only with culture medium and irradiated (irradiation control) or incubated with compounds and not exposed to radiation (non-irradiated control). After 1 h, the cells were fixed in 4% paraformaldehyde with 1% sucrose for 15 min at room temperature, permeabilized with 1% Triton X-100, and blocked with 6% bovine serum albumin for 20 min. Cells were incubated overnight at 4 °C with a 1:100 dilution of anti-phospho-histone γH2AX (Ser139, 20E3) antibody and anti-53BP-1 rabbit mAb. After incubation with the primary antibodies, cells were washed with PBS and incubated with a 1:500 dilution of fluorophore-conjugated secondary antibodies for 1 h at room temperature. DAPI staining was performed to visualize cell nuclei. Images were captured using an Olympus IX81 fluorescence microscope (Olympus America, Melville, New York) and analyzed with ImageJ. The number of γ-H2AX–positive foci and the fluorescence intensity of 54BP-1-stained cells were quantified to assess treatment-induced DNA DSBs.

### Biodistribution in normal mice

Biodistribution studies were carried out in conformity with Portuguese law according to the EU Guidelines for Animal Care and Ethics in Animal Experimentation by properly accredited researchers and were approved by the national authority, Direção-Geral da Alimentação e Veterinária (DGAV), and the local animal welfare committee. The animals were housed in a temperature and humidity-controlled room with a 12 h light/dark schedule in animal house facilities approved by DGAV.

The biodistribution of the ^111^In-M-Carb was evaluated in 2 groups of healthy female CD1 mice (Charles River, France) weighing between 29 and 36 g each. The animals were intravenously injected with 100 μL (2.8–3.7 MBq) of the preparation via the tail vein and were maintained on a normal diet ad libitum. Mice were euthanized by cervical dislocation at 1 h and 24 h post-injection. The radioactive administered dose and the radioactivity in the sacrificed animal were measured in a dose calibrator (Carpintec CRC55tW). The difference between the radioactivity in the injected and sacrificed animal was assumed to be due to excretion and expressed as a percentage of the total injected activity. Tissue samples of the main organs were then removed, rinsed, and weighed, then radioactivity was counted in a gamma counter (HIDEX AMG, Hidex). The uptake in the organs/tissues was calculated and expressed as a percentage of the injected activity per organ (% I.A.).

After radioactive decay, the major organs were also analyzed by ICP-MS to assess the boron content. Thus, main organs were freeze-dried and digested in medium pressure HP500 vessels with a mixture of HNO_3_, H_2_O_2_ and H_3_PO_4_ (Merck pro analysis, USA) using a closed pressurized microwave digestion unit (Mars5, CEM, USA) at 90 °C for 5 h. After cooling, the samples were diluted with ultrapure water and the boron content measured by a Thermo X-Series Quadrupole ICP-MS (Thermo Scientific, USA), as a service provided by Laboratório Central de Análises at Universidade de Aveiro.

### Statistical analysis

Data were analyzed by using GraphPad Prism version 10 for Windows. Unpaired t tests were used to compare differences between survival metrics or foci numbers. Two-way ANOVA followed by Tukey’s post hoc test was used to analyze colony numbers. Levels of statistical significance were denoted as follows: not significant, *p* > 0.05; *p < 0.05; **p < 0.01; ***p < 0.001.

## Supplementary Material

Supporting Info_ uploaded to NIHMS on Oct 5 2026

The online version contains supplementary material available at https://doi.org/10.1186/s12645-026-00400-2.

Supplementary Material 1.

## Figures and Tables

**Fig. 1:**
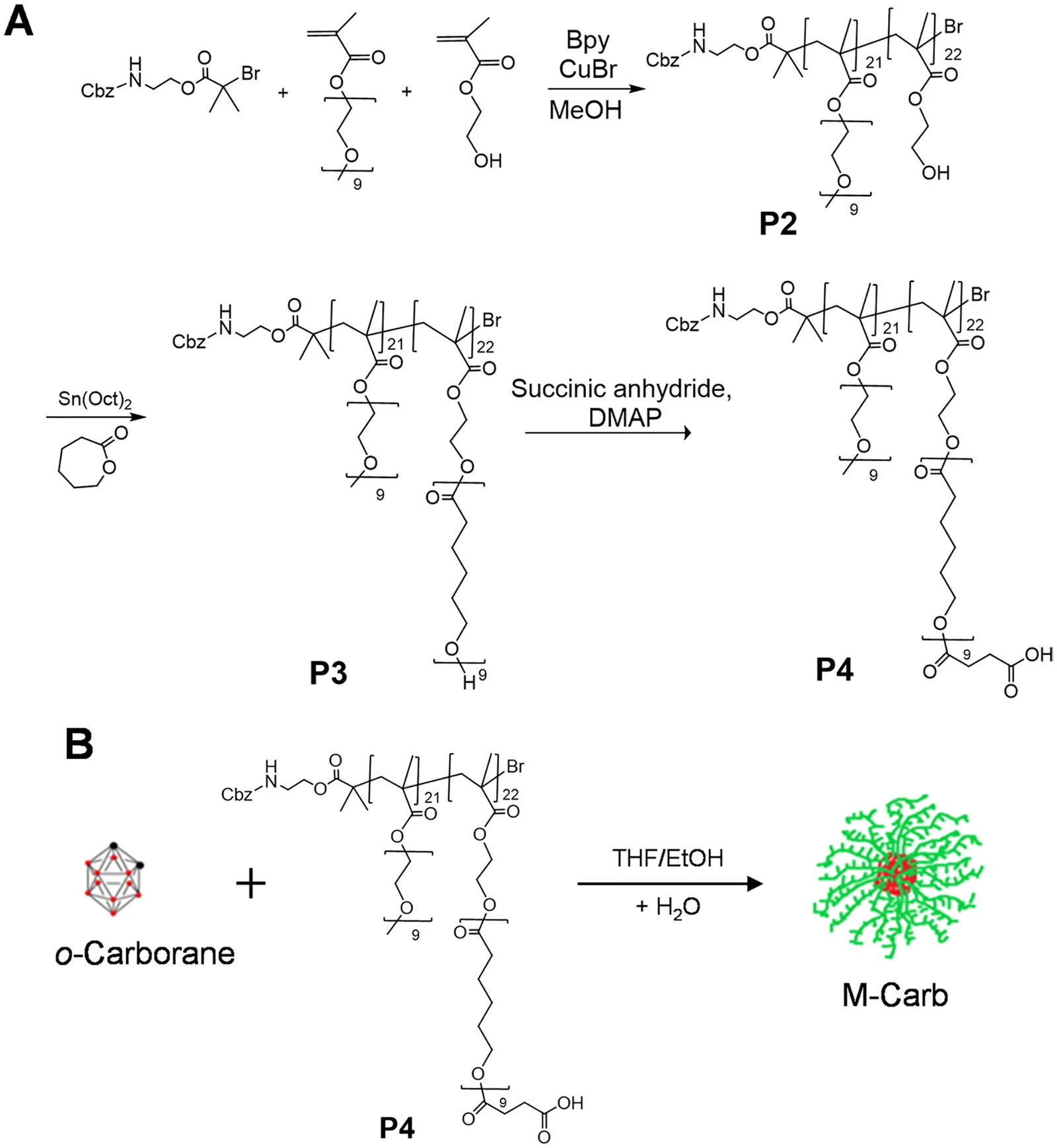
Synthesis of copolymer P4 **A** and formulation of *o*-carborane-entrapped polymeric micelles (M-Carb) **B**. The micelles were self-assembled by adding water to tetrahydrofuran (THF) solution of polymer mixed with *o*-carborane in ethanol (EtOH). Bpy, 2,2’-bipyridine; Sn(Oct)_2_, stannous octoate; DMAP, dimethyl aminopyridine

**Fig. 2:**
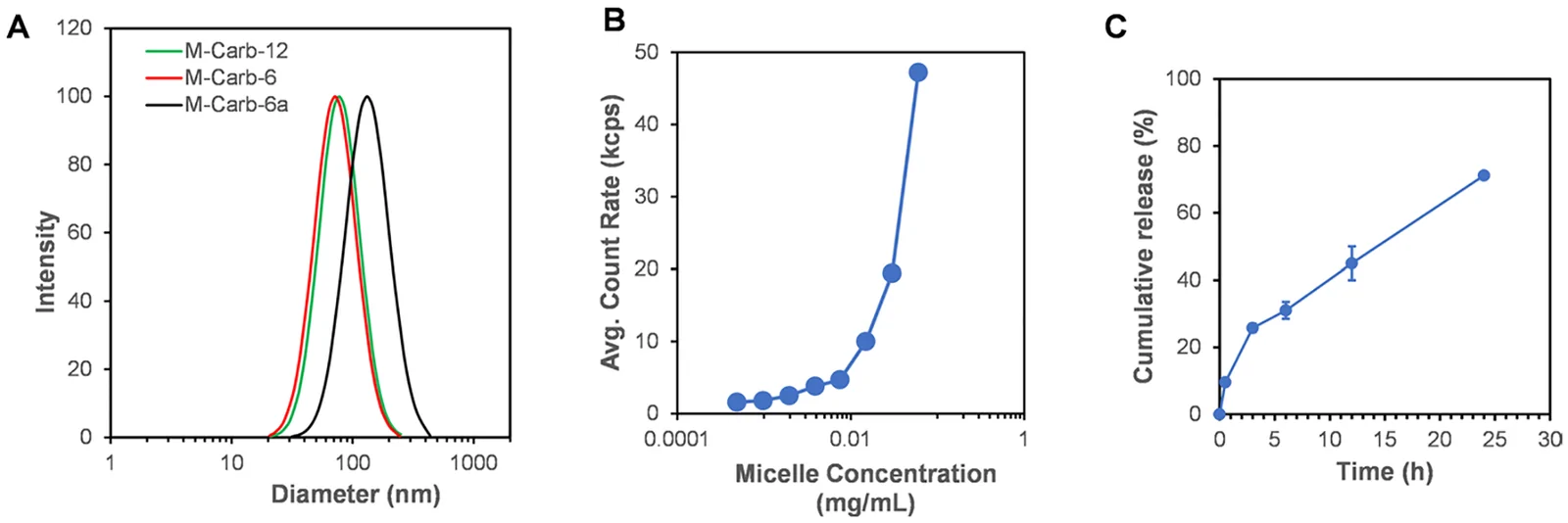
Characterization of M-Carb. **A** Size and size distribution of M-Carb determined by dynamic light scattering (DLS). M-Carb-6 and M-Carb-12 were prepared using the THF/ethanol system. M-Carb-6a was prepared using the THF system. Details are provided in Table 1. Micelles were dispersed in PBS at a concentration of 1 mg P4/mL for the measurement. **B** Critical micelle concentration measured by DLS in PBS for M-Carb-12. **C***o*-Carborane release from M-Carb-12 at 37 °C in PBS containing 10% FBS. *o*-Carborane was quantified by measuring boron concentration by ICP-OES. Data are expressed as mean ± SD (*n* = 3)

**Fig. 3:**
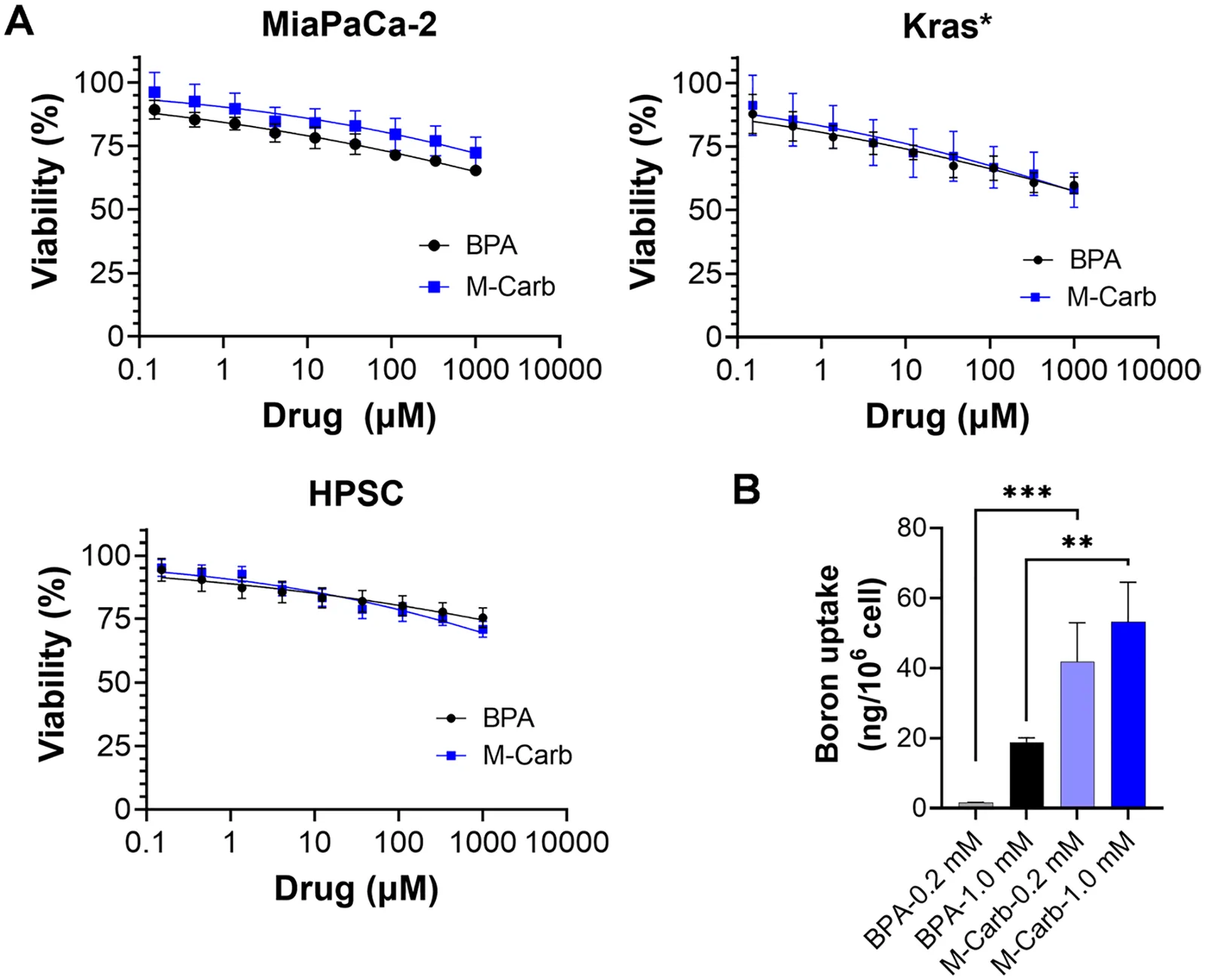
In vitro cytotoxicity and cellular uptake. **A** Cytotoxicity of M-Carb in comparison with BPA in 3 cell lines. Relative cell viability was normalized to the viability value of the untreated control measured at 72 h by the CCK-8 assay. **B** Uptake of M-Carb and BPA in MiaPaCa-2 cells. Cells were incubated with M-Carb and BPA at 37 °C for 6 h. Data are expressed as mean ± SD (*n* = 3–5). Boron concentration was measured by ICP-MS. **p < 0.01, ***p < 0.001 (Student’s t test)

**Fig. 4:**
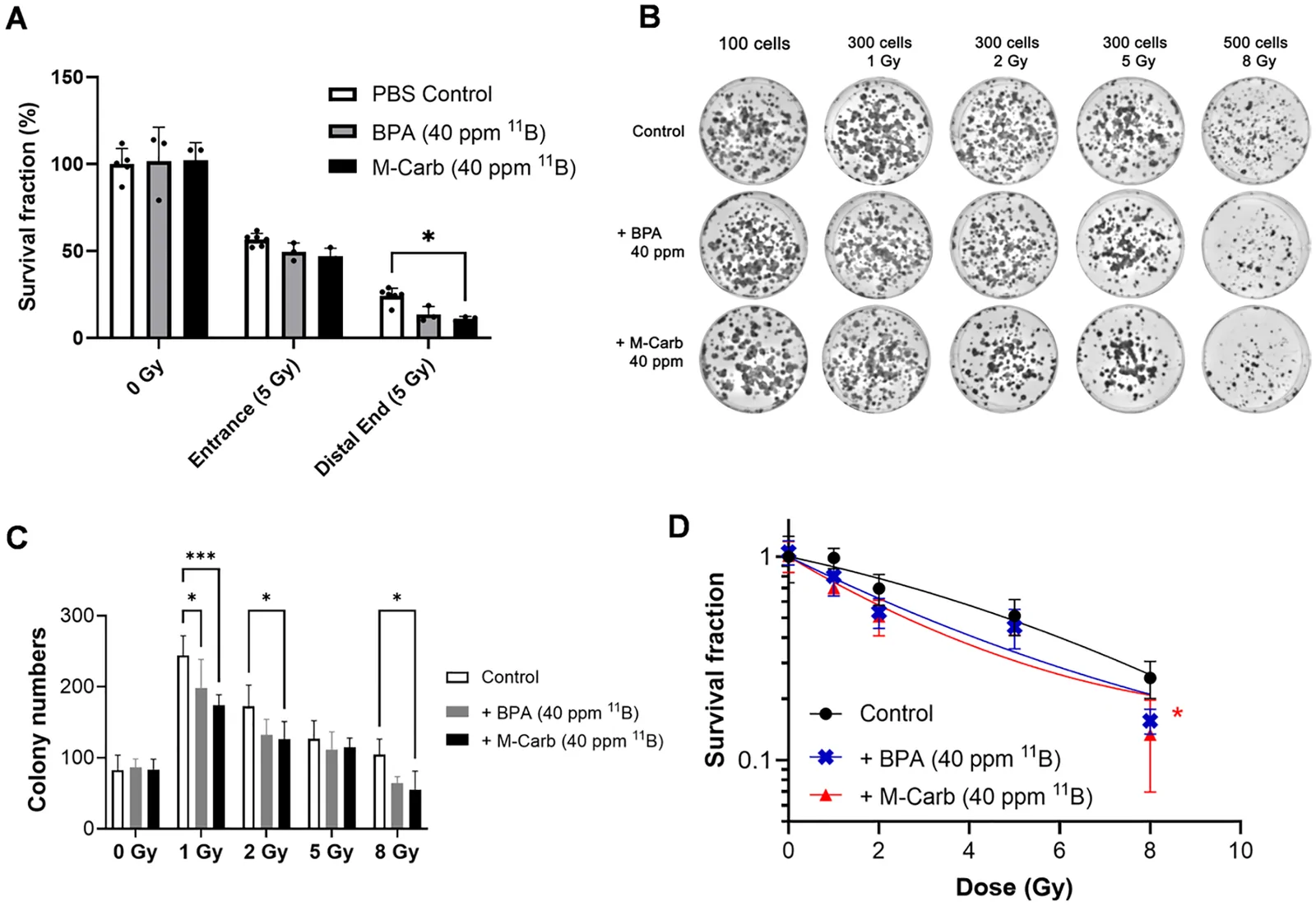
Colony formation assay demonstrating radiosensitizing effects of boron-containing compounds M-Carb and BPA in human MiaPaCa-2 PDAC cells. **A** Effect of proton irradiation LET. MiaPaCa-2 cells were incubated with PBS control, BPA with 40 ppm equivalent ^11^B, and M-Carb with 40 ppm equivalent ^11^B for 6 h. After washing steps, cells were either not irradiated or irradiated with a 5 Gy proton beam at entrance (low LET) or distal end (high LET) spread-out Bragg peak. Data are expressed as mean ± SD (*n* = 3–6). *p < 0.05 (Student t test). **B** Representative photographs of cells in 6-well plates in clonogenic assay showing effect of radiation dose. MiaPaCa-2 cells were treated with PBS, 40 ppm BPA, or 40 ppm M-Carb with no irradiation or with proton beam irradiation at the distal end at the indicated radiation doses. After 14 days of incubation, cells were stained with crystal violet and the number of colonies were counted. **C** Quantitative analysis of the number of colonies. Data are presented as mean ± SD (*n* = 6 for the control group, *n* = 3 for the BPA and M-Carb groups). * *p* < 0.05, *** *p* < 0.001 (two-way ANOVA followed by Tukey’s post hoc test). **D** Analysis of survival fraction. *p < 0.05 (two-way ANOVA followed by Tukey’s post hoc test)

**Fig. 5:**
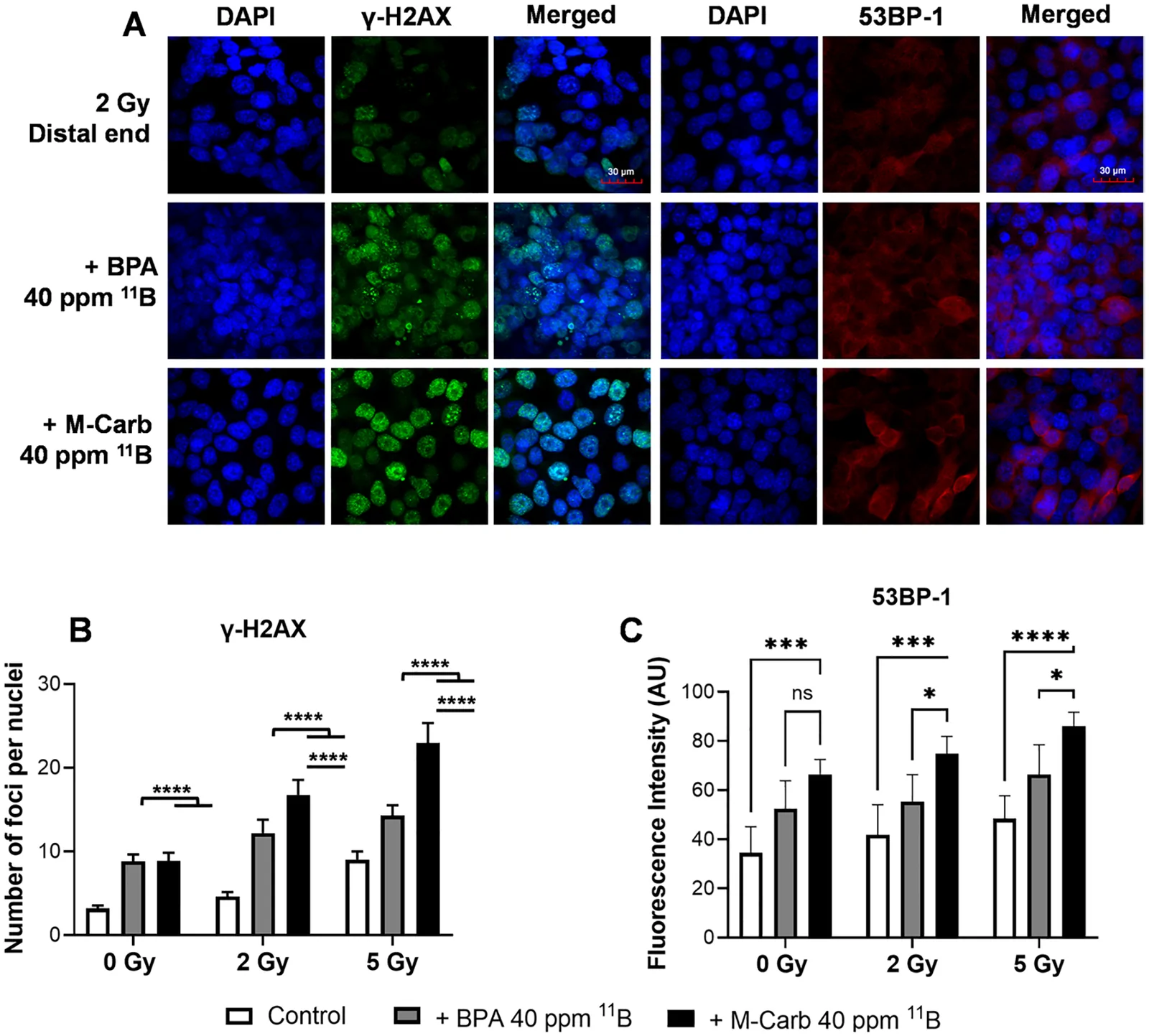
Confocal imaging of MiaPaCa-2 cells stained with γ-H2AX and 53BP1. **A** Representative fluorescence micrographs of cells treated with 2-Gy proton irradiation in the presence and absence of boronated compounds. Random fields from a representative experiment out of 2 independent experiments are shown. Scale bar = 30 μm. **B** Quantification of the number of γ-H2AX foci as an indication of DNA double-strand breaks (DSBs). Data indicate mean ± SD. ****p < 0.0001 (*n* = 4/group). **C** Bar graph representation of fluorescence intensity of 53BP-1-stained cells in arbitrary units (AU) in the absence or presence of BPA or M-Carb and irradiated at the indicated proton beam doses. *p < 0.05, ** *p* < 0.01, and *** *p* < 0.001 *n* = 4/group (two-way ANOVA followed by Tukey’s post hoc test)

**Fig. 6:**
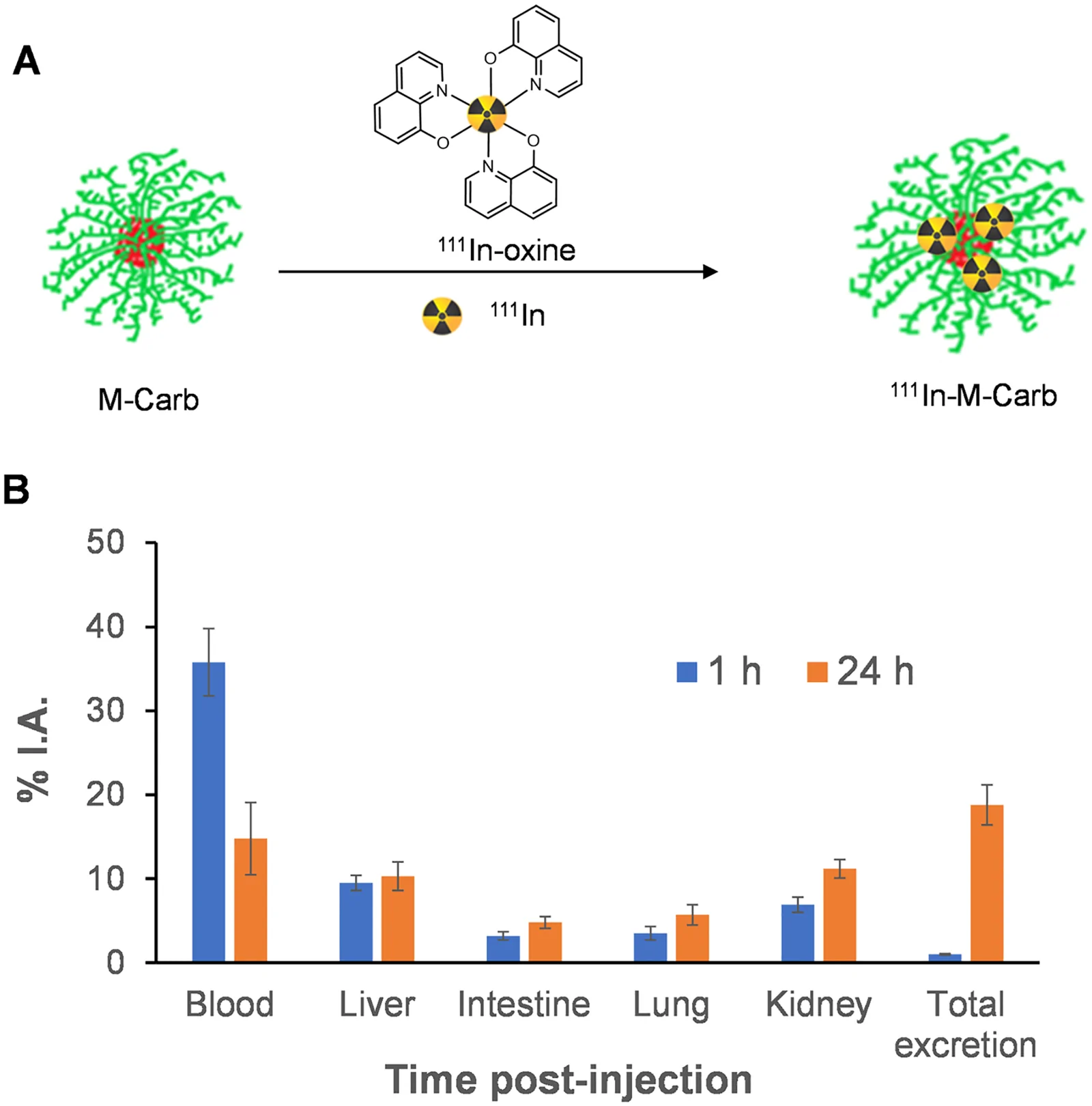
Biodistribution of ^111^In-labeled M-Carb. **A** Schematic drawing of the ^111^In labeling of the M-Carb micelles. A micelle aqueous solution was treated with ^111^In-oxine to afford the radiolabeled micelles ^111^In-M-Carb in high yield (ca. 90%). **B** Biodistribution data in most relevant organs. CD1 mice were intravenously injected with ^111^In-M-Carb, and ex vivo biodistribution studies were performed at 1 and 24 h post-injection by measurement of the radioactivity in different organs and tissues

**Table 1:** Preparation and characterization of M-Carb*

Batch	P4 in THF (mg/mL)	Carb Conc. (mg/mL)	THF/ EtOH Ratio (v: v	Feed Ratio P4:Carb	EE (%)	LC (%)	Size (nm)	PDI	Zeta (mV)
M-Carb-6	100	25^a^	1:0.24	100:6	65.3	3.77	73.3 ± 0.1	0.112	−8.3 ± 2.4
M-Carb-12	100	25^a^	1:0.48	100:12	57.5	6.45	76.4 ± 0.7	0.080	−8.0 ± 3.0
M-Carb-6a	100	25^b^	1:0	100:6	58.0	3.36	131.5 ± 0.3	0.157	−8.1 ± 1.4
M-Carb-6b	50	12.5^a^	1:0.24	100:6	56.3	3.30	74.4 ± 0.2	0.110	−8.7 ± 2.5
M-Carb-12a	50	12.5^a^	1:0.48	100:12	52.8	6.00	71.7 ± 0.2	0.160	−9.1 ± 1.3

* Polymer solution: Surface charge was measured in 10 mM PBS. EE: entrapment efficiency; LC: loading capacity; PDI, polydispersity index. Drug: *o*-carborane.

a Solvent-EtOH;

b Solvent-THF. Data are expressed as mean ± SD (*n* = 3)

## Data Availability

No datasets were generated or analysed during the current study.
