# Supplementary material for: o-carborane encapsulated into polymeric micelles for proton boron capture therapy
Source: Cancer Nanotechnol. Author manuscript; Available in PMC 2026 Oct 20. (PMC13679845; doi:10.1186/s12645-026-00400-2)
Supplement: Supplementary file 1 [file NIHMS2214758-supplement-Supporting_Info__uploaded_to_NIHMS_on_Oct_5_2026.pdf]

Supporting Information

Table S1. Survival fraction analysis using the linear quadratic regression model.

	**Control**	**+ BPA (40 ppm)**	**+ M-Carb (40 ppm)**
α	0.109 ± 0.103	0.251 ± 0.113	0.302 ± 0.122
β	0.007 ± 0.018	-0.007 ± 0.019	-0.013 ± 0.020
α/β	15.015	-35.572	-22.821
R^2	0.787	0.864	0.842
$D_{50\%}$(Gy)	4.849	3.015	2.582

Table S2. ***Ex vivo*** **biodistribution and excretion data of ^{111}In-M-Carb in CD1 mice at 1 and 24 h post-injection***

Organ	1 h	24 h
**Blood**	35.8 ± 4.0	14.8 ± 4.3
**Liver**	9.5 ± 0.9	10.3 ± 1.7
**Intestine**	3.2 ± 0.5	4.8 ± 0.7
**Spleen**	0.6 ± 0.2	0.8 ± 0.2
**Heart**	0.49 ± 0.04	0.6 ± 0.2
**Lung**	3.5 ± 0.8	5.7 ± 1.2
**Kidney**	6.9 ± 0.9	11.2 ± 3.1
**Muscle**	16.8 ± 2.7	11.2 ± 3.2
**Bone**	8.1 ± 0.5	8.7 ± 0.7
**Stomach**	0.51 ± 0.02	0.37 ± 0.06
**Pancreas**	0.31 ± 0.06	0.29 ± 0.05
**Excretion**	1.0	18.8 ± 4.4

*Results are expressed as a percentage of the injected activity per organ or tissue (% I.A.) assuming that total mass of blood, bone and muscle, correspond to 6, 10 and 40% of body weight, respectively. Excretion result is expressed as a percentage of the total injected activity (% I.A.) and was calculated by the difference between the radioactivity in the injected and sacrificed animal.

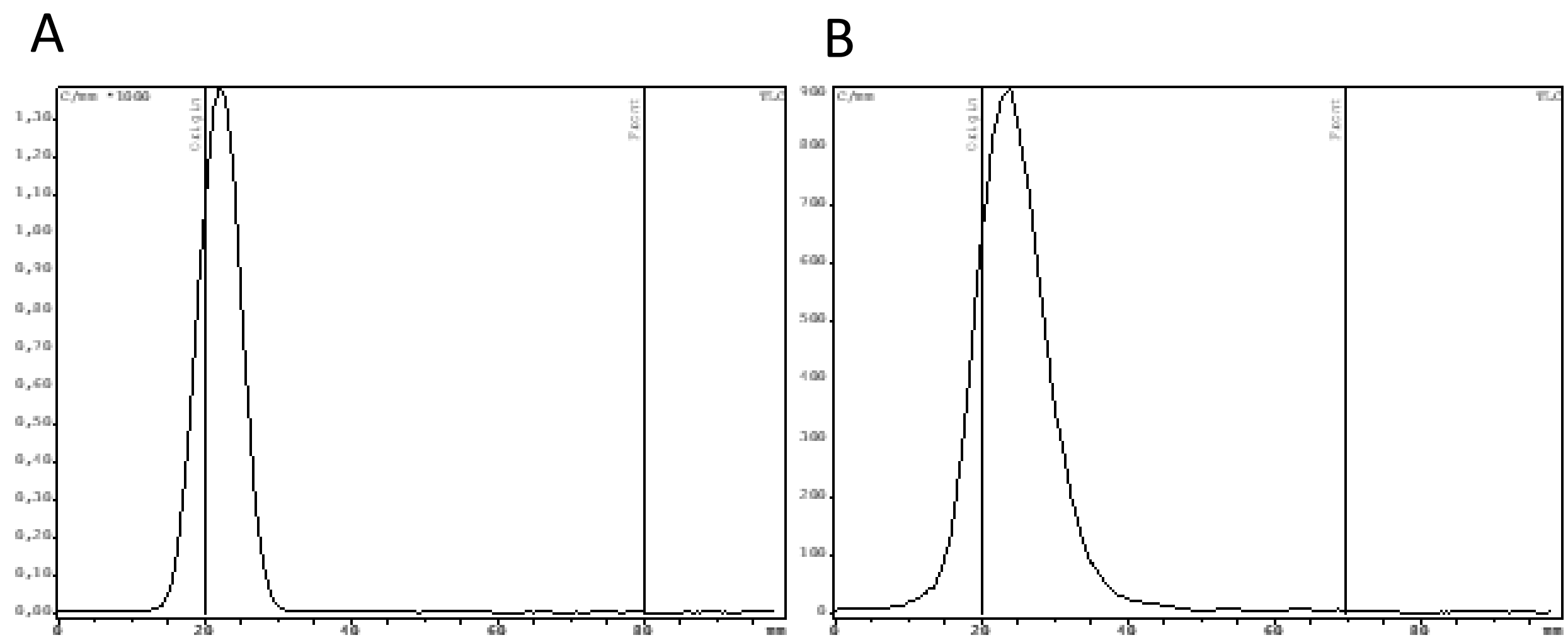

Figure S1. Radio-chromatograms of labeled micelles ^{111}In-M-Carb after purification (A) and after incubation in 0.1 M PBS solution pH 7.4 at 37 °C for 24 h (B). Chromatographic system: ITLC-SG using sodium acetate buffer pH 5.0 as eluent.
